# Simultaneous DHA and organic selenium production by *Schizochytrium* sp.: a theoretical basis

**DOI:** 10.1038/s41598-023-42900-w

**Published:** 2023-09-20

**Authors:** Yunqiang Zhang, Zikui Liu, Gang Xiao, Jiawei Shi, Baili Liu, Ning Xiao, Zhiliang Sun

**Affiliations:** 1 Hunan Agricultural University Veterinary Faculty, No.1 Nongda Road, Furong District, Changsha City, 410000 Hunan China; 2 Hunan Canzoho Biological Technology Co., Ltd., 321 Kangning Road, Changsha City, 410000 Hunan China

**Keywords:** RNA, Bioinformatics, Applied microbiology

## Abstract

Docosahexaenoic acid (DHA) and selenium (Se) are nutrients that confer several health benefits to both humans and animals. Widespread use of DHA in milk powder and health products requires large-scale mass production via *Schizochytrium* sp., while Se intended for human consumption is produced as organic Se via yeast. However, producing these nutrients on an industrial scale is constrained by various factors. We found that supplementing *Schizochytrium* sp. with Na_2_SeO_3_ (0.5 mg/L) improves its biomass and DHA production and also provides organic Se. De novo assembled transcriptome and biochemical indicators showed that Na_2_SeO_3_ promotes forming acetyl coenzyme A and L-cysteine via the glycerol kinase and cysteine synthase pathways, promoting DHA synthesis through the polyketide synthase pathway. However, high doses of Na_2_SeO_3_ (5 mg/L) limited the biomass of *Schizochytrium* sp. and DHA content. This study provided a theoretical basis for the simultaneous production of organic Se and DHA via *Schizochytrium* sp.

## Introduction

*Schizochytrium* spp. are unicellular, spherical marine fungi considered important biological resources. These species are commonly used to industrially produce docosahexaenoic acid (DHA) because of their stable growth, rapid division, and high content of DHA. DHA is an important long-chain polyunsaturated fatty acid found in the brain, liver, nerve tissues, and other organs of mammals. DHA promotes human brain development during infancy and improves intelligence^1^. Birch et al.^2^ found that adding DHA to infant formula can improve the future visual development of term infants. DHA also affects amyloid proteins and the blood–brain barrier carrier protein, APOɛ-4, alleviating the high incidence of Alzheimer’s disease in the elderly^3,4^. DHA reduces blood lipid levels and lowers the risk of cardiovascular diseases^5^. Furthermore, DHA plays a neuroprotective role by affecting the cell membrane thickness via acyl groups, the formation of which involves phospholipids, cell receptors, pathways, and peripheral proteins. Therefore, DHA may be considered indispensable to the human body. The factors and mechanisms that influence DHA production by *Schizochytrium* spp. have been discussed previously. The fatty acid synthase (FAS) pathway is not a major pathway involved in DHA synthesis in *Schizochytrium* spp; the synthesis of DHA in *Schizochytrium* sp. is catalyzed via the polyketide synthase (PKS) pathway rather than relying on carbon-chain-elongation reactions catalyzed by dehydrogenation or desaturation enzymes^6^. This finding provides a new method for studying the mechanisms underlying DHA production in *Schizochytrium* spp. However, the specific synthetic mechanisms and factors influencing DHA synthesis remain unclear.

Selenium (Se) is an important trace element in the human body, and its deficiency may cause many diseases. Due to the scarcity of Se on land, it must be supplied via food to meet worldwide demand^7^. Although organic Se produced by yeast is a high-quality source of Se that the human body can use, its use is limited by high production costs^8^. The utilization of Se by microorganisms is crucial for circulating and transforming Se forms in nature, indicating that Se plays a vital role in microbial metabolism^9^. Although excessive Se inhibits the growth and metabolism of microorganisms, some microorganisms can tolerate moderate amounts of Se^10^. In theory, the ability of *Schizochytrium* spp. to simultaneously produce DHA and organic Se may enable it to be used in a low-cost, high-value-added production system. Because DHA is present in oil, and organic Se is present in proteins, the two can be separated using simple processing technology. Although only a few studies have researched this production mode, it may allow full use of the resources and reduce costs, qualifying it as a potentially widely used method.

## Experimental procedures

### Materials

*Schizochytrium* sp. 20,888, available from the US Strain Collection (ATCC), and stored in ATCC using + 790 medium at − 80 °C was used; the culture medium (basic fermentation medium) involved Dextrose 90g/l (Qiqihar Longjiang Fufeng Biotechnology Co., Ltd.). Yeast powder 12g/l (Beijing Hongrun Baoshun Technology Co., Ltd.), Milk peptone 10g/l (Beijing Hongrun Baoshun Technology Co., Ltd.), Sea salt 20g/l (Jinan Zichen Chemical Co., Ltd.) were used. Detection kits for malondialdehyde (MDA), glutathione peroxidase (GSH-Px), acetyl coenzyme A (A-CoA), and reactive oxygen species (ROS) were purchased from Nanjing Jiancheng Biotechnology Co., Ltd. De novo transcriptome assembly was performed by BGI Genomics Co., Ltd.

### Design

The sample was randomly divided into five groups: C, T1, T2, T3, and T4, using a single-factor design. Group C (the control group) was cultured in a basic fermentation medium, while groups T1, T2, T3, and T4 were treated with low (0.1 mg/L), medium (0.5 mg/L), high (1 mg/L), and ultra-high (5 mg/L) doses of sodium selenite (Na_2_SeO_3_) based on the C group, respectively, with three parallel replications being performed in each group. After 72 h of fermentation, the liquid was dried via centrifugation (4400 × *g*) and stored in liquid nitrogen for the de novo transcriptome assembly and detection of other indicators. The indicators targeted included the biomass, DHA content, and Se content, and the levels of MDA, GSH-Px, A-COA, and ROS.

## De novo transcriptome assembly

### Introduction to transcriptome methods

Briefly, the procedure involved the following: (1) Total RNA was processed via mRNA enrichment or rRNA removal; enrichment of mRNA where mRNA with PolyA tail was enriched using magnetic beads with oligodt; (2) Removal of rRNA, where rRNA was hybridized with a DNA probe, DNA/RNA hybridization chain was selectively digested using RNaseH, and the DNA probe was digested with DNaseI, to obtain the required RNA after purification; (3) the obtained RNA was fragmented using interrupt buffer and reverse transcribed with random N6 primers, following which the cDNA double strand was used to form double stranded DNA; (4) The end of the synthesized double stranded DNA was flattened and phosphorylated at the 5’ end, whence the 3’ end formed a sticky end protruding an “a”, which was connected to a bubbly joint with a protruding “T” at the 3’ end; (5) The linked products were amplified via PCR using specific primers; (6) The PCR product was thermally denatured into a single strand, and then a bridge primer was used to cyclize the single strand DNA to obtain a single strand circular DNA library; and (7) computer sequencing was performed.

### Sequencing data analysis method

First, low-quality reads with joint contamination and a high content of unknown base N were filtered out. Filtered data were considered clean reads. Clean reads were assembled to obtain UniGene, using which functional annotation and SSR detection were performed. Next, we calculated the expression level of each sample based on UniGene, detected differentially expressed genes (DEGs) between the different samples, annotated the genetic function of the sequencing results with reference to the seven databases, and performed an in-depth cluster analysis and a functional enrichment analysis of the DEGs.

### Quantitative real-time PCR (RT-qPCR)

The RNA extraction kit was purchased from Omega Bio Tek (Code No. R6840). Reverse transcription and RT-qPCR kits were purchased from Takara Bio Inc (Code No. RR047A and RR820A), and the experimental steps were strictly carried out according to the instructions. Briefly, the total RNA of the sample was extracted and reverse transcribed into cDNA, following which the cDNA was diluted. RT-qPCR amplification was conducted to verify the efficiency of primer amplification, which was used as a reference gene to normalize expression levels between samples. Primers were designed using Primer 5. The 2^-ΔΔCt^ method was used to characterize the differential expression multiple of genes. The Ct value was defined as the number of cycles required for the fluorescence signal of the amplification product to reach a set threshold during the PCR process.

### Detection and analysis of biochemical indicators

MDA levels were determined using the thiobarbituric acid method^11^. GSH-Px was measured using the 5,5’-dithiobis-(2-nitrobenzoic acid) method (DTNB); the concentration of GSH in the reaction system decreasing by 1 μmol/L was an enzyme activity unit (AU)^12^. The ROS level was measured using the 2’,7'-dichlorodihydrofluorescein diacetate method, and fluorescence intensity was read by a microplate reader for relative comparison (Arbitrary Unit, AU)^13^. A-CoA was measured using a double-antibody sandwich enzyme-linked immunosorbent assay^14^. These tests were performed according to the manufacturer’s instructions. Biomass was estimated as the ratio of cell dry matter weight to fermentation broth weight.

### DHA content detection

The DHA content in the fermentation broth was determined via gas chromatography using the detection principle described by Alinafiah et al.^15^. Sample pretreatment: (1) lipid extraction: a quantitative amount of *Schizochytrium* spp. (dry matter) was weighed and added to the tubes. Anhydrous ethanol (10 mL), anhydrous ether (25 mL), and petroleum ether (25 mL) were added, shaken, mixed well, and transferred to a ground flat-bottom flask; the process was repeated thrice. Next, the solvent was rotated and evaporated at 50 °C to near dryness; (2) Saponification and esterification: the concentrate obtained from (1) was added to a methanol solution of 0.5 mol/L potassium hydroxide, refluxed on a water bath at 70 °C for 10 min, and treated with 5 mL of a 13% mass fraction boron trifluoride methanol solution. After refluxing for 10 min, the mixture was transferred to a centrifuge tube. Next, 10 mL of *n*-hexane was added as the internal standard, the mixture was centrifuged (4400 × *g*), and the supernatant was passed through anhydrous sodium sulfate for testing.

### Se content detection

The Se content of the samples was determined via hydride generation atomic fluorescence spectrometry. Sample processing and operating conditions described by Sun and Feng^16^ were followed. Briefly, (1) the pretreatment for inorganic Se detection: first, 0.1 g of the obtained *Schizochytrium* sp. dry powder was accurately weighed and treated with 10 mL of 3 mol/L hydrochloric acid. After disrupting the ultrasonic walls for 5 min, the mixture was placed in a boiling water bath for 10 min, centrifuged (17,700 × *g*) for 10 min, and the supernatant was collected; these steps were repeated twice, and the supernatants were merged. (2) The pretreatment for total Se detection: the precipitate obtained from (1) was treated with 20 mL nitric acid/perchloric acid mixture (4/1, v/v). After mixing the treated mixture with the supernatant, it was slowly heated on an electric heating plate until it was nearly dry and dissolved in 5 mL of HCl at a concentration of 6 mol/L for testing. (3) The samples obtained from steps (1) and (2) were detected using atomic fluorescence spectrophotometer to determine the content of inorganic Se and total Se, respectively. The organic Se content was calculated by subtracting the inorganic Se content from the total Se content.

### Data processing and analysis

The obtained data were preliminarily sorted and analyzed using Excel. Statistical analyses were performed using SPSS 22.0. An ANOVA was used for comparing statistical differences, and Tukey’s method was used for multiple comparisons. The results were expressed as mean ± standard deviation, and *P* < 0.05 was considered a significant difference.

## Results and discussion

### Biomass, DHA, and Se content

**Figure 1:**
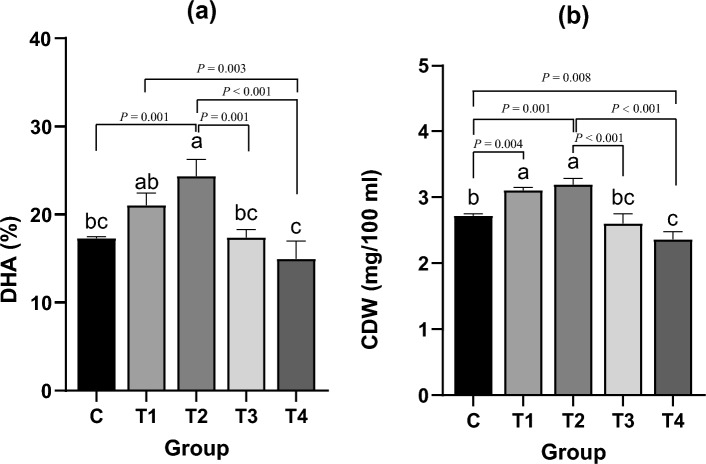
Effect of supplying Na_2_SeO_3_ on fermentation results. Different lowercase letters “abc” represent significant differences between different treatments of same data (*P* < 0.05).

Changes in the biomass and DHA content of *Schizochytrium* sp. under different Na_2_SeO_3_ supplementation doses showed consistency. Compared to group C, groups T1 and T2 showed an increase in biomass (group T1 and T2 significantly increased by 14.34% and 17.28%, respectively; *P* = 0.004 and *P* = 0.001) and DHA content (group T2 significantly increased 40.74%, *P* = 0.001), while groups T3 and T4 did not show a significant promoting effect, but an inhibition trend instead (Fig. 1). These findings indicated that the biomass and DHA content of *Schizochytrium* sp. were promoted at appropriate concentrations of Na_2_SeO_3_ but inhibited by higher or increased Na_2_SeO_3_ concentrations.

**Figure 2:**
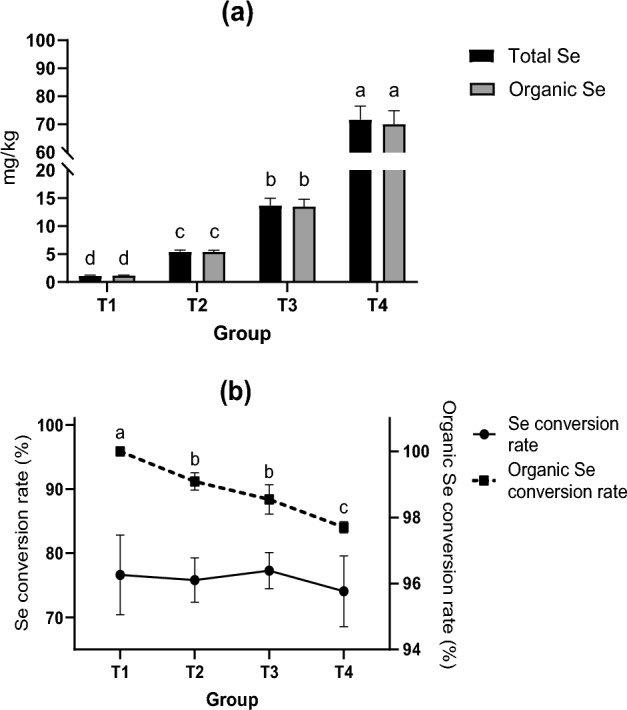
Effect of supplying Na_2_SeO_3_ on Se content. Different lowercase letters “abc” represent significant differences between treatments for the same data (*P* < 0.05).

The total Se and organic Se in *Schizochytrium* sp. Increased significantly (*P* < 0.05) due to supplementation with Na_2_SeO_3_ in a dose-dependent manner (Fig. 2). However, there were no statistically significant differences between the Se conversion rates.

1 $${\text{Se conversion rate}} = {\text{ total Se }} \times {\text{ cell dry matter weight }}/{\text{ added Se}}$$

where added Se is calculated based on mass fraction of Se in Na_2_SeO_3_;

2 $${\text{Organic Se conversion rate }} = {\text{ organic Se }}/{\text{ total Se}}.$$

Se was not detected in control group C, so it is not shown here.

The ratio of organic Se in *Schizochytrium* sp. also showed a downward trend, wherein that of group T1 was significantly higher than that of the other groups; meanwhile, that of the T4 group was significantly lower than those of the T2 and T3 groups (*P* = 0.001 and *P* = 0.021). The results showed that, although the content of total Se and Organic Se increased with increasing Na_2_SeO_3_ during the fermentation process of *Schizochytrium* sp., the utilization efficiency of organic Se decreased. The organic Se conversion rate in group T1 was 100%. There may be two possible reasons for this result: (1) due to the low content of Na_2_SeO_3_ (0.1 mg/L) supplementation in group T1, the level of inorganic selenium was lower than the detection limit. Therefore, the organic selenium content was calculated to be 100%. (2) The utilization rate by *Schizochytrium* sp. may be too high for the small amount of Na_2_SeO_3_ involved, resulting in almost all of it being converted to organic Se.

### Biochemical indicators

**Figure 3:**
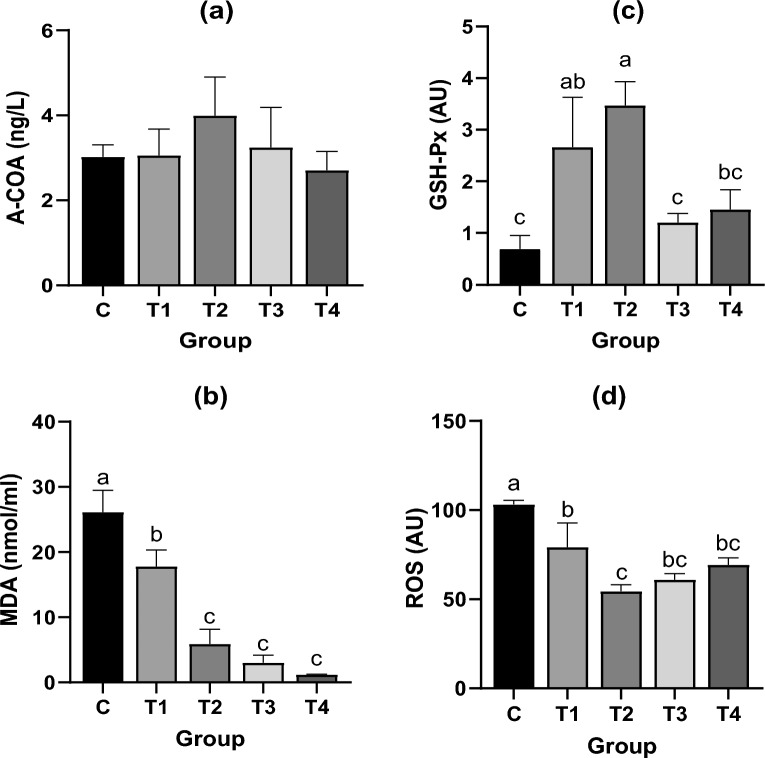
Comparison of A-COA, MDA, GSH-Px, and ROS levels in each group. Different lowercase letters “abc” represent significant differences between different treatments of same data (*P* < 0.05).

The biochemical test results for each group showed a certain regularity (Fig. 3). Although group T2 was slightly higher than the other groups in A-COA, there was no significant difference. As the concentration of Na_2_SeO_3_ increased, the MDA levels decreased in a dose-dependent manner, and the MDA levels in all treatment groups were significantly lower than those in group C (the significance of groups T1, T2, T3, and T4 were *P* = 0.001, *P* < 0.001, *P* < 0.001 and *P* = 0.001). As the Na_2_SeO_3_ concentration increased, GSH-Px activity first increased and then decreased. GSH-Px activity in the T1 and T2 groups was significantly higher (*P* = 0.007 and *P* = 0.001) than in group C. However, the level of ROS in group T2 was significantly lower than that in T1 (*P* = 0.008), and the level in group T1 was significantly lower (*P* < 0.05) than that in group C (*P* = 0.010).

### RNA-seq and de novo assembly

The raw sequencing data contained low-quality reads, joint contamination, and a high content of unknown bases. To ensure the reliability of the results, these reads were removed before data analysis. See Table S1 for the quality index of filtered reads. The clean reads ratio of each group was greater than 90%, guaranteeing its reliability. Trinity was used to assemble clean reads and cluster the transcripts to obtain UniGene. TIGR Gene Indices clustering tools were used to cluster the UniGene of each sample again to get the final UniGene, named “all UniGene,” for subsequent analysis (Fig. S1).

### Annotation of gene function

To obtain more comprehensive information on gene function, we annotated the longest transcript with reference to seven databases (NR, NT, Pfam, GO, KOG, SwissProt, and KEGG). Five databases (NR, NT, KOG, KEGG, and GO) with similar research purposes and high annotation success rates were selected to draw a Venn diagram (Fig. S2). Although 3474 genes could be correctly annotated in the five databases, many could not. This low annotation was attributed to two factors: (i) some transcriptome data consisted of noncoding sequences, and (ii) *Schizochytrium* spp. are not well studied, with only a few notes regarding this species and related species being found in existing databases.

### Differentially expressed genes (DEGs)

Deseq2 was used to analyze and detect the DEGs between samples according to the gene expression level of each sample. The screening threshold for differential genes was Padj < 0.05 and | log2foldchange |> 1. If the log2 fold-change of the gene was > 0, that DEG was considered upregulated; otherwise, the DEG was considered downregulated.

**Figure 4:**
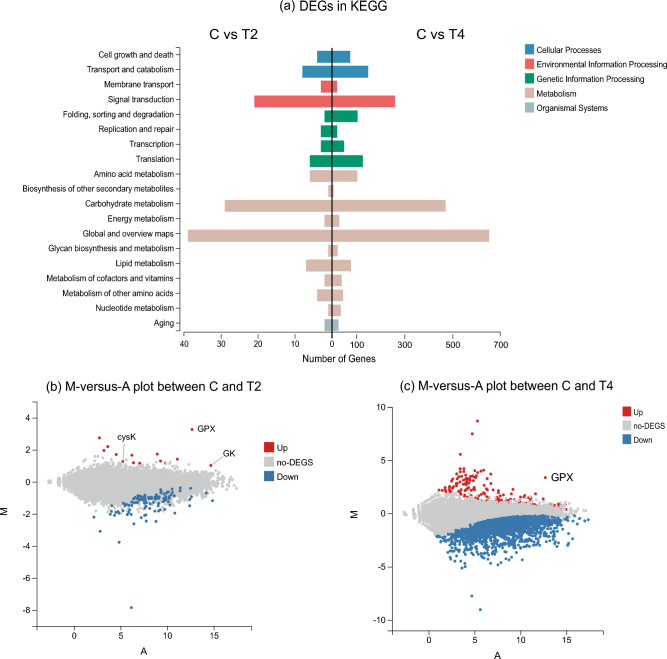
Classification of DEGs in KEGG (**a**) and M-versus-A plot of DEGs in T2 and T4 (**b** and **c**). In M-versus-A plot, X-axis represents A (average log2 gene expression), and Y-axis represents M (log2 fold change). Color red represents upregulated DEGs, color blue represents downregulated DEGs, and color gray represents non-DEGs.

Group T2, the key research group here, showed a large increase in DHA and biomass compared with group C. In contrast, group T4 showed a large degree of inhibition compared with group C, attributed to the inhibition effect exerted by a high dose of Na2SeO3, which was used in a negative regulation study. The pathway research of this study is based on the KEGG database^17–19^. The differential pathway classifications of the DEGs between groups C, T2, and T4 in the KEGG database are shown (Fig. 4a). The supplementation of *Schizochytrium* sp. with Na_2_SeO_3_ may affect transcriptional expression in different pathways, and the number of DEGs in group T4 group was much higher than that in group T2. These results indicated that moderate concentrations of Na_2_SeO_3_ (T2) increased the biomass and DHA content by affecting the DEGs of *Schizochytrium* sp. In contrast, excessive Na2SeO3 (T4) concentrations produced more complex changes in transcriptional expression, resulting in inhibitory effects.

According to the DEGs M-versus-A plots of the T2 and T4 groups (Fig. 4b,c), the T4 group exhibited more DEGs than the T2 group, with a significantly higher proportion of downregulated genes. Based on the research direction, gene expression characteristics, and results, eight representative genes highly likely to be associated with the results were selected for verification via real-time q-PCR (Table 1).

**Table 1:** Significantly enriched pathway terms in KEGG and primer sequences for RT-qPCR.

Gene Name	Definition	Primer sequences
β-acting	House-keeping genes	F: AGACAAGAACTACGAGCTTC
		R: GTCTACATCGCACTTCATGA
thyA	Thymidylate synthase	F: CGTTGGTAGAGCTGACACGA
		R: GTAGCCTCGCAAATGTACTC
MNN5	alpha 1,2-mannosyltransferase	F: CAGAAAGCCATCTCCATGT
		R: CGAACTCTCCTTCATCAGTATC
GPX	Peroxiredoxin	F: AGCTCGAGGTGCTTCTGTTC
		R: AGCGTGCAGTTGGAGATGAA
cysK	Cysteine synthase	F: GTCGACGATGAGAACAACAG
		R: CTGTTTCGCCAGCTTTCT
PIGW	Glucosaminylphosphatidylinositol acyltransferase	F: CCCCACTTTCGCCCAAAAC
		R: AGGCAGGGCTAGTGTAGTGT
GK	Glycerol kinase	F: GATGCTCTCTTCGGTACCAT
		R: AGGGTCTTGATGTCCATGAG
THOC2	THO complex subunit 2	F: GGAAACAACCGCTCCCAAAC
		R: TTGATGTTGGTGTTGCTGCG
SNQ2	ATP-binding cassette, subfamily G	F: GCCATCAACGTGTCCTTT
		R: GGAAGCCGAAATCGAGATAC

**Figure 5:**
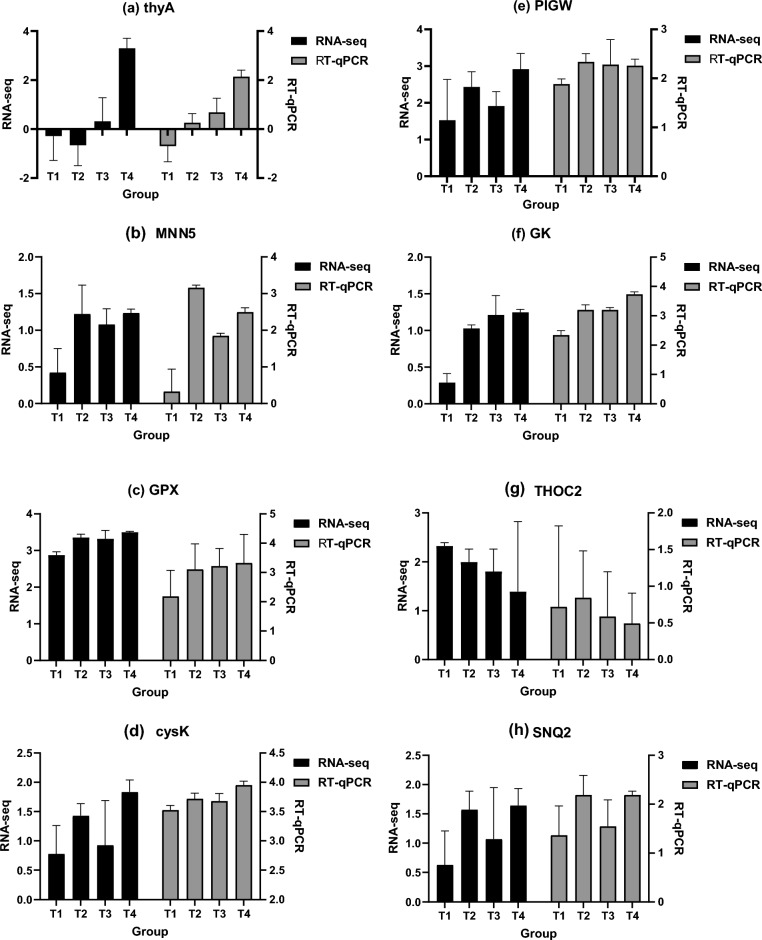
Comparison of RNA-seq and RT-qPCR results of gene mRNA expression level.

To verify the reliability of transcriptome results, the relative expression difference multiple of the selected genes was calculated using Group C as the reference; the RNA-seq was calculated via log2Ratio, and the RT-qPCR was calculated via −ΔΔCt. The gene expression trends of RNA-seq and RT-qPCR were similar, indicating that the transcriptome sequencing results were reliable (Fig. 5).

### Glycerol kinase (GK) pathway

The GK pathway plays an important role in glycerol metabolism. The results showed that the GK expression in group T2 was significantly higher than in group C (Fig. 5f). *Schizochytrium* sp. utilizes glucose to generate glycerol via glycolysis and glycerol 3-phosphate (G3P) via GK to generate dihydroxyacetone phosphate (DHAP)^20^. DHAP and glyceraldehyde triphosphate oscillate between each other through the action of triose phosphate isomerase, although they are restricted to some extent by the dose concentration^21^. Glyceraldehyde triphosphate generates pyruvate via glyceraldehyde kinase and 3-phosphate glycerate mutase^22^. In organisms, pyruvate is converted into A-COA, NADH, and CO_2_ via the pyruvate dehydrogenase complex^23^. In the PKS pathway, A-COA is a substrate necessary for DHA synthesis^24^. Therefore, supplementing a *Schizochytrium* sp. culture medium with Na_2_SeO_3_ can promote the synthesis of A-COA via the GK pathway, thereby promoting the synthesis of DHA via the PKS pathway^25^. However, although the A-COA content in the T2 group was slightly higher than that in the other groups, the difference was insignificant (Fig. 3a). This may be due to the participation of A-COA in many biochemical reactions simultaneously, such as the tricarboxylic acid cycle, causing its content to undergo complex dynamic changes^26^, thereby preventing the difference in COA content from being detected by this test.

Additionally, a metabolite of the GK pathway, G3P, may synergize with the glycerol promoter. An experiment exploring halophiles found that glycerol kinase activity is regulated by the concentration of environmental glycerol metabolites^27^. This indicated that an increase in glycerol concentration may enhance the expression of glycerol kinase and that the glycerol promoter is linked to an increase in the efficiency of glycerol phosphorylation^28^. The DHA present in *Schizochytrium* spp. has a triglyceride structure, and DHAP and G3P, as important precursors for glycerol synthesis, directly affect the synthesis of glycerol esters^29,30^. Therefore, promoting the GK pathway plays an important role in forming the triglyceride DHA here.

### Cysteine synthase (cysK) pathway

Selenium is a trace element that is essential for the growth and metabolism of most microorganisms. Usually, microorganisms cannot process Na_2_SeO_3_ directly and thus convert Na_2_SeO_3_ into selenophosphate via selenophosphate synthetase SelD/SPS2 to initiate antioxidant functions^31^. Increased cysK expression, thus, increased L-cysteine production (Fig. 5d). The results of the DEG analysis showed that peroxiredoxin (GPX) was significantly expressed in the T2, T3, and T4 groups (Fig. 5c). This suggests that in the presence of Na_2_SeO_3_, *Schizochytrium* spp. utilize cysteine, glutamic acid, and glycine to produce glutathione (GSH). GSH then forms GSH-Px with the aid of glutathione reductase (GR)^32^. The addition of an appropriate amount of Na_2_SeO_3_ to the fermentation broth increased GSH-Px activity and significantly reduced (*P* < 0.05) ROS and MDA levels (Fig. 3b–d). Large amounts of ROS, which destroy cell integrity via the removal of electrons, are produced during the metabolism and proliferation of *Schizochytrium* spp.^33^. Additionally, ROS oxidize DHA synthesized by *Schizochytrium* sp. to MDA, reducing the production of DHA and attenuating growth^34^. This study proved that the supplementation of *Schizochytrium* sp. with an appropriate amount of Na_2_SeO_3_ promotes the expression of cysK, increases the activity of GSH-Px, and reduces the ROS and MDA levels, which is closely associated with increases in *Schizochytrium* sp. biomass and DHA content.

PKS is the main pathway associated with DHA synthesis in *Schizochytrium* spp.^6^. The route of this pathway is composed of β-ketoacyl synthetase, β-ketoyl-ACP reductase, enoyl reductase, dehydratase/isomerase, acyltransferase, and acyl carrier protein, which enable its completion. In the PKS pathway, A-COA and malonyl-CoA are used as substrates and undergo condensation, reduction, dehydration, and isomerization cycles, ultimately forming DHA^35^. The type II PKS pathway consists of a constant minimum PKS core, including a malonyl acyltransferase, an acyl carrier protein (ACP), and an α/β Isodimer ketone synthase (KS α-KS β), with the smallest PKS core dominating the main functions of this approach^36,37^, wherein, KS α plays an assembly role^38,39^. Bräuer et al.^40^ studied the type II PKS pathway using X-ray crystallography. They confirmed the presence of a flexible cysteine binding site (cys176) in KSα, which enables the flexible cysteamine group to transfer the malonyl building block from 4'-phosphopantetheine to the ketosynthases in cys176. Here, the cysteine synthase expression increased, which increased the synthesis of L-cysteine. Thus, we deduced that using cysteine by KSα in the PKS pathway improved and promoted DHA synthesis.

### Inhibitory effect of excessive Na_2_SeO_3_

When the Na_2_SeO_3_ supplement reached 1 mg/L, the biomass and DHA content of *Schizochytrium* sp. began to decline, and the growth itself was inhibited when Na_2_SeO_3_ reached 5 mg/L (Fig. 1b). As Na_2_SeO_3_ continued to be added, the utilization rate of Se by *Schizochytrium* sp. showed a downward trend (Fig. 2b). This indicated that a high dose of Na_2_SeO_3_ could not be fully utilized by *Schizochytrium* sp. Nine significantly downregulated DEGs that may inhibit the growth and metabolism of *Schizochytrium* spp. were identified via screening (Table 2). Oxalate-CoA ligase participates in the tricarboxylic acid cycle^41^. Acyl-CoA oxidase is closely related to forming substrates for fatty acid synthesis^42^. Furthermore, 2,4-dienoyl-CoA reductase participates in polyunsaturated fatty acid β oxidation^43^. Acetyl-CoA synthetase is closely associated with the production of cellular triglycerides^44^. Acyl-CoA dehydrogenase plays an important role in mitochondrial metabolism^45^. The fermentation broth of *Schizochytrium* sp. is a high-salt environment, and choline dehydrogenase plays an important role in salt tolerance^46^. Signaling mucin MSB2 promotes the filamentous growth of yeast cells, indicating its association with cell division^47^. Glutamate dehydrogenase is a common catalytic enzyme in glutamic acid metabolism^48^. Aldehyde dehydrogenase reduces the toxic effects of ethanol metabolism by producing acetaldehyde^49^. The expression of these key enzymes plays a role in the growth and metabolism of *Schizochytrium* spp. Thus, a decreased expression of these enzymes may account for the lowering of its biomass and DHA content. However, judging by the number of DEGs, the reasons for the inhibitory effects exerted by an excess of Na_2_SeO_3_ on *Schizochytrium* spp. are complicated, and further investigations were warranted.

**Table 2:** Significantly downregulated differential genes that may inhibit growth and metabolism of *Schizochytrium* sp.

Gene name	Definition in KEGG	log_2_FoldChange	*P*-value
AAE3	Oxalate-CoA ligase [EC:6.2.1.8]	−2.844070	0.000079
E1.3.3.6, ACOX1, ACOX3	acyl-CoA oxidase [EC:1.3.3.6]	−2.288498	0.003075
DECR2, SPS19	2,4-dienoyl-CoA reductase [(3E)-enoyl-CoA-producing], peroxisomal [EC:1.3.1.124]	−2.355674	0.002388
ACSS1_2, acs	acetyl-CoA synthetase [EC:6.2.1.1]	−2.808455	0.000084
ACADM, acd	acyl-CoA dehydrogenase [EC:1.3.8.7]	−2.576526	0.000002
betA, CHDH	choline dehydrogenase [EC:1.1.99.1]	−3.751287	2.781186e-7
MSB2	signaling mucin MSB2	−2.446892	0.000114
E1.4.1.4, gdhA	glutamate dehydrogenase (NADP +) [EC:1.4.1.4]	−2.602952	0.000007
ALDH	aldehyde dehydrogenase (NAD +) [EC:1.2.1.3]	−2.435926	0.000540

Log_2_FoldChange refers to multiple gene differences between T4 and C groups. Among selected genes, difference between T4 and C was significant, whereas difference between T2 and C was not.

Interestingly, although the expression level of GPX significantly increased in groups T3 and T4 (Fig. 5c), GSH-Px activity was significantly lower than that in group T2 (*P* < 0.05) (Fig. 3c). In contrast, there was no significant difference between the levels of MDA and ROS in these groups (Fig. 3b and d). When microorganisms are exposed to selenite (SeO_3_^2−^) toxicity, mercaptan can react with SeO_3_^2−^ and achieve detoxification^50^. GSH is a mercaptan commonly found in *Schizochytrium* spp. GSH reacts with SeO_3_^2−^ to generate selenium-glutathione (GS-Se-SG), which is further reduced to GS-Se^-^ via the action of GR and thioredoxin reductase (TR), and hydrolyzed to GSH and se^0^, during which process many ROS are generated^51^. This indicates that with an excess of Na_2_SeO_3_, GSH will consume GR to detoxify SeO_3_^2−^ by acting as a “catalyst” by changing se^-4^ to red se^0^, which prevents GSH from converting to GSH-Px, and limits its ability to remove ROS and MDA.

It was observed that the fermentation broth of group T4 was redder than that of group C. We confirmed that there was a large amount of se^0^ in the fermentation broth of the T4 S*chizochytrium* sp. The ratio of organic Se in group T4 decreased significantly (*P* < 0.05), indicating that the ratio of inorganic Se (se^0^) in *Schizochytrium* sp. had increased; this finding was consistent with the above inference (Fig. 2). During this process, selenium was mostly present in the form of selenoamino acids, which explained why the organic selenium content still significantly increased even though high doses of sodium selenite had inhibitory effects (Fig. 1).

## Conclusion

**Figure 6:**
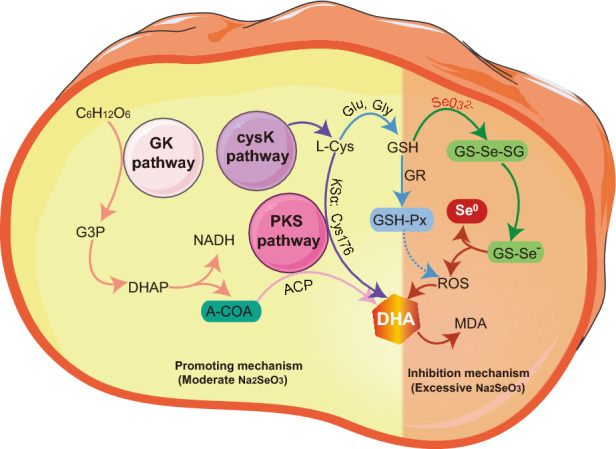
Schematic diagram of mechanism of Na_2_SeO_3_ effect on *Schizochytrium* sp.

The mechanism of the effect of Na_2_SeO_3_ on *Schizochytrium* sp. is shown in Fig. 6. Adding an appropriate amount of Na_2_SeO_3_ (0.5 mg/L) during the fermentation process of S*chizochytrium* sp. promotes the growth of its biomass and DHA content. This growth is attributed to promoting GK and cysK pathways and generating GSH-Px. However, any further increase in the Na_2_SeO_3_ concentration (1 and 5 mg/L) lowers the biomass and DHA content of *Schizochytrium* sp. The conversion to organic Se may be achieved using various levels of Na_2_SeO_3_ supplementation; however, increasing the amount of Na_2_SeO_3_ would decrease the conversion rate of organic Se due to its toxic effects. Thus, this study provides the theoretical basis for a new method to simultaneously enhance the DHA content and produce organic Se, using S*chizochytrium* sp. In any actual production process, the proportion of Na_2_SeO_3_ supplementation may be adjusted based on the economic benefits expected.

### Supplementary Information

Supplementary Information.

## Data Availability

The datasets generated and/or analysed during the current study are available in the NCBI-SRA database, [https://www.ncbi.nlm.nih.gov/sra/PRJNA1002230].
