# Supplementary material for: Simultaneous DHA and organic selenium production by Schizochytrium sp.: a theoretical basis
Source: Sci Rep. 2023 Sep 20;13:15607. doi: 10.1038/s41598-023-42900-w (PMC10511486; doi:10.1038/s41598-023-42900-w)
Supplement: Supplementary file 1 — Supplementary Information. [file 41598_2023_42900_MOESM1_ESM.pdf]

Table S1. Read quality statistics

Sample	Total Raw Reads (M)	Total Clean Reads (M)	Total Clean Bases(Gb)	Clean Reads Q20(%)	Clean Reads Q30(%)	Clean Reads Ratio(%)
C1	45.44	43.16	6.47	97.53	92.69	94.99
C2	45.44	43.53	6.53	97.63	92.89	95.80
C3	43.69	42.02	6.30	97.63	92.92	96.19
T11	45.44	43.44	6.52	97.78	93.31	95.61
T12	45.44	43.37	6.51	97.73	93.19	95.44
T13	45.44	43.23	6.48	97.97	93.70	95.14
T21	45.44	43.24	6.49	97.98	93.69	95.16
T22	45.44	42.40	6.36	97.91	93.62	93.31
T23	43.69	42.34	6.35	97.81	93.24	96.90
T31	43.69	42.09	6.31	97.77	93.15	96.35
T32	43.69	42.14	6.32	97.92	93.55	96.45
T33	43.69	42.02	6.30	98.03	93.84	96.18
T41	45.44	43.58	6.54	97.91	93.50	95.90
T42	45.44	43.28	6.49	97.90	93.52	95.25
T43	45.44	43.47	6.52	97.91	93.58	95.68

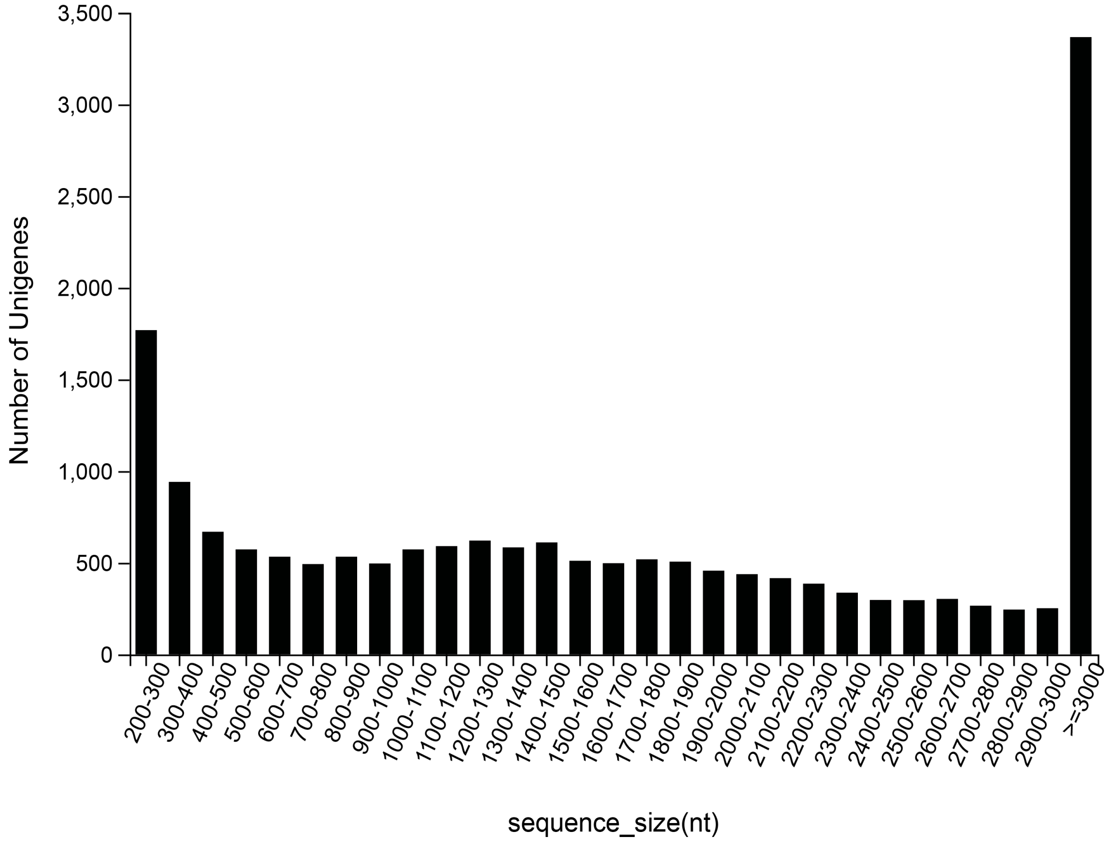

Fig. S1 Unigene length distribution

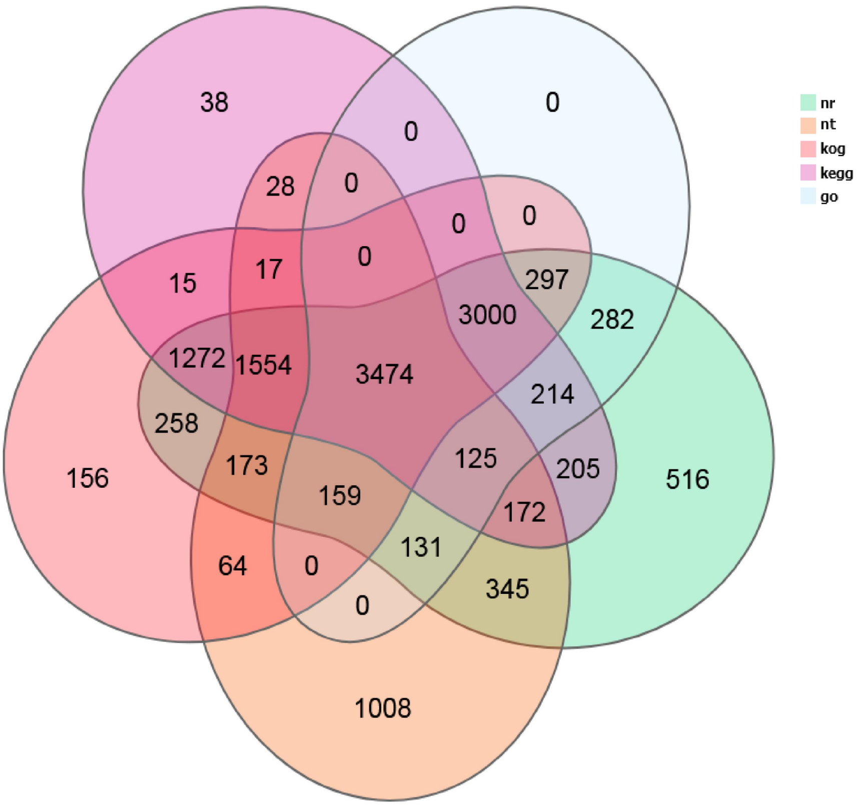

Fig. S2. Venn diagram of the gene function annotation results.
